# Botanical Drug Extracts Combined With Biomaterial Carriers for Osteoarthritis Cartilage Degeneration Treatment: A Review of 10 Years of Research

**DOI:** 10.3389/fphar.2021.789311

**Published:** 2022-01-31

**Authors:** Panyun Mu, Jie Feng, Yimei Hu, Feng Xiong, Xu Ma, Linling Tian

**Affiliations:** ^1^ Department of Clinical Medicine, Chengdu University of Traditional Chinese Medicine, Chengdu, China; ^2^ Department of Orthopedics, Hospital of Chengdu University of Traditional Chinese Medicine, Chengdu, China

**Keywords:** osteoarthritis, botanical drug, extracts, cartilage, tissue engineering, biomaterial carriers

## Abstract

Osteoarthritis (OA) is a long-term chronic arthrosis disease which is usually characterized by pain, swelling, joint stiffness, reduced range of motion, and other clinical manifestations and even results in disability in severe cases. The main pathological manifestation of OA is the degeneration of cartilage. However, due to the special physiological structure of the cartilage, once damaged, it is unable to repair itself, which is one of the challenges of treating OA clinically. Abundant studies have reported the application of cartilage tissue engineering in OA cartilage repair. Among them, cell combined with biological carrier implantation has unique advantages. However, cell senescence, death and dedifferentiation are some problems when cultured *in vitro*. Botanical drug remedies for OA have a long history in many countries in Asia. In fact, botanical drug extracts (BDEs) have great potential in anti-inflammatory, antioxidant, antiaging, and other properties, and many studies have confirmed their effects. BDEs combined with cartilage tissue engineering has attracted increasing attention in recent years. In this review, we will explain in detail how cartilage tissue engineering materials and BDEs play a role in cartilage repair, as well as the current research status.

## 1 Introduction

Osteoarthritis (OA) is a progressive arthrosis disease (Crivelli et al., 2019), the main pathophysiological features of which include cartilage defects, synovitis, osteophyte formation, and subchondral bone damage (Yeh et al., 2015; Wang et al., 2018). OA patients suffer from joint pain and stiffness, difficulty in movement, and ultimately disability (Wang et al., 2018; Jin, 2020), which significantly affects their quality of life (Rahimi et al., 2021), especially for the elderly (Kann et al., 2016), and places a huge economic burden on their families and society (Wang et al., 2018; Jin, 2020). According to the World Health Organization, more than 10% of people under the age of 60 worldwide suffer from OA (Wang et al., 2018). By 2032, the number of people over the age of 45 suffering from OA will increase from 25 to 29%, and the peak of incidence will be around the age of 75 (Qin et al., 2020).

### 1.1 Ultrastructure of Bone and Joint, and Pathogenesis of OA

In a joint, the articular cartilage, calcified cartilage, subchondral cortex, and trabecular bone form biological complexes called “osteochondral units” (Goldring and Goldring, 2016). The articular cartilage is mainly composed of extracellular matrix (ECM) and chondrocytes (1–2%). The ECM consists mainly of Type II collagen (COL2), glycosaminoglycan (GAG), aggrecan (ACAN), elastin fibrils, and 70% water (Goldring and Goldring, 2016; Jin, 2020) and provides tensile and elastic force for the articular cartilage (Crivelli et al., 2019) to maintain the proper biomechanical function of the joint. The development, maintenance, and repair of the ECM are controlled by chondrocytes derived from highly specialized and metabolically active mesenchymal stem cells (MSCs). The shape, number, and size of chondrocytes, stiffness of the cartilage, ECM composition, and content of proteoglycan, COL2, and water in the ECM vary with the anatomical regions of the articular cartilage (Rai et al., 2017)

Articular cartilage can be damaged by daily wear and abnormal mechanical load (Xia et al., 2014). With the progression of OA, a disintegrin and metalloproteinase with thrombospondin motifs 5 (ADAMTS5) and matrix metalloproteinase 13 (MMP13) are the main enzymes causing cartilage damage (Wang et al., 2013; Miller et al., 2016; Jin et al., 2020a). In the early stages of OA, inflammatory stimuli induce the cartilage and synovial cells to secrete these enzymes. Steoarthritic chondrocytes mediate the production of inflammatory mediators, including interleukin 1 (IL-1), tumor necrosis factor (TNF), prostaglandins, and nitric oxide (NO) through the nuclear factor-kappa B (NF-κB) signaling pathway (Charlier et al., 2019). IL-1β and TNF-α are effective inducers of matrix metalloproteinases (MMPs). Among them, proteases MMP1, MMP3, and MMP13 inhibit proteoglycan and collagen synthesis, mediate chondrocyte apoptosis, and promote cartilage inflammation (Abramson, 2008; Blanco et al., 2011; Oliveira Silva et al., 2020).

### 1.2 Treatment Status of Osteoarthritis

The lack of vascular and aneural tissue in articular cartilage gives it a limited intrinsic self-repair ability (Sheu et al., 2013; Lima et al., 2019), which makes the treatment of articular cartilage defects an extremely difficult clinical problem (Makris et al., 2015). The current methods include conservative and nonconservative treatments. The conservative treatments include non-pharmacologic, pharmacologic, and alternative therapies (Madry et al., 2011; Kang et al., 2020), while nonconservative therapeutic strategies mainly include bone marrow stimulation (Frehner and Benthien, 2018), autologous or allogeneic osteochondral transplantation (Bugbee et al., 2016; Di Martino et al., 2021), autologous chondrocyte implantation, and periosteum transplantation (Mistry et al., 2017). Although such treatments have been widely used in clinical practice, they still have obvious and inevitable limitations and deficiencies (Vinatier and Guicheux, 2016; Xu et al., 2020).

Drug therapies include nonsteroidal anti-inflammatory drugs (NSAIDs), opioids, and glucocorticoids. Most therapies are limited to symptomatic treatment aiming at relieving pain and improving joint function rather than inhibiting the progression of OA (Jin, 2020). However, the long-term use of such drugs can cause serious adverse reactions such as gastrointestinal reactions and osteoporosis (Crivelli et al., 2019; Shi et al., 2019). Beyond oral administration, other routes have been developed for the treatment of OA. Different from other diseases, OA is limited to one or more joints, which offers a special opportunity for local intra-articular (IA) drug injection. IA allows the delivery of therapeutic drugs directly to the diseased joint at very high concentrations; moreover, it limits the absorption of drugs into the systemic circulation, which reduces systemic toxicity. Thus, compared with the systemic approach, a smaller dose can achieve a therapeutic effect similar to that of oral administration (Burt et al., 2009; Jones et al., 2019). IA injections of hyaluronic acid (HA) or glucocorticoids have been used to relieve knee OA (KOA) pain (Berenbaum et al., 2012). However, the main limitation of IA injection is the rapid elimination of the drug from the articular cavity. Studies have shown that small-molecule drugs (MW < 10 kDa) can be removed from the synovial fluid by lymphatic drainage within 5 h of injection (Zhang et al., 2018; Jones et al., 2019), and thus multiple injections are required, which can lead to infection or joint disability. To control the release rate over a long period of time, a new biology-based drug delivery vector is needed (Chen et al., 2012).

**FIGURE 1:**
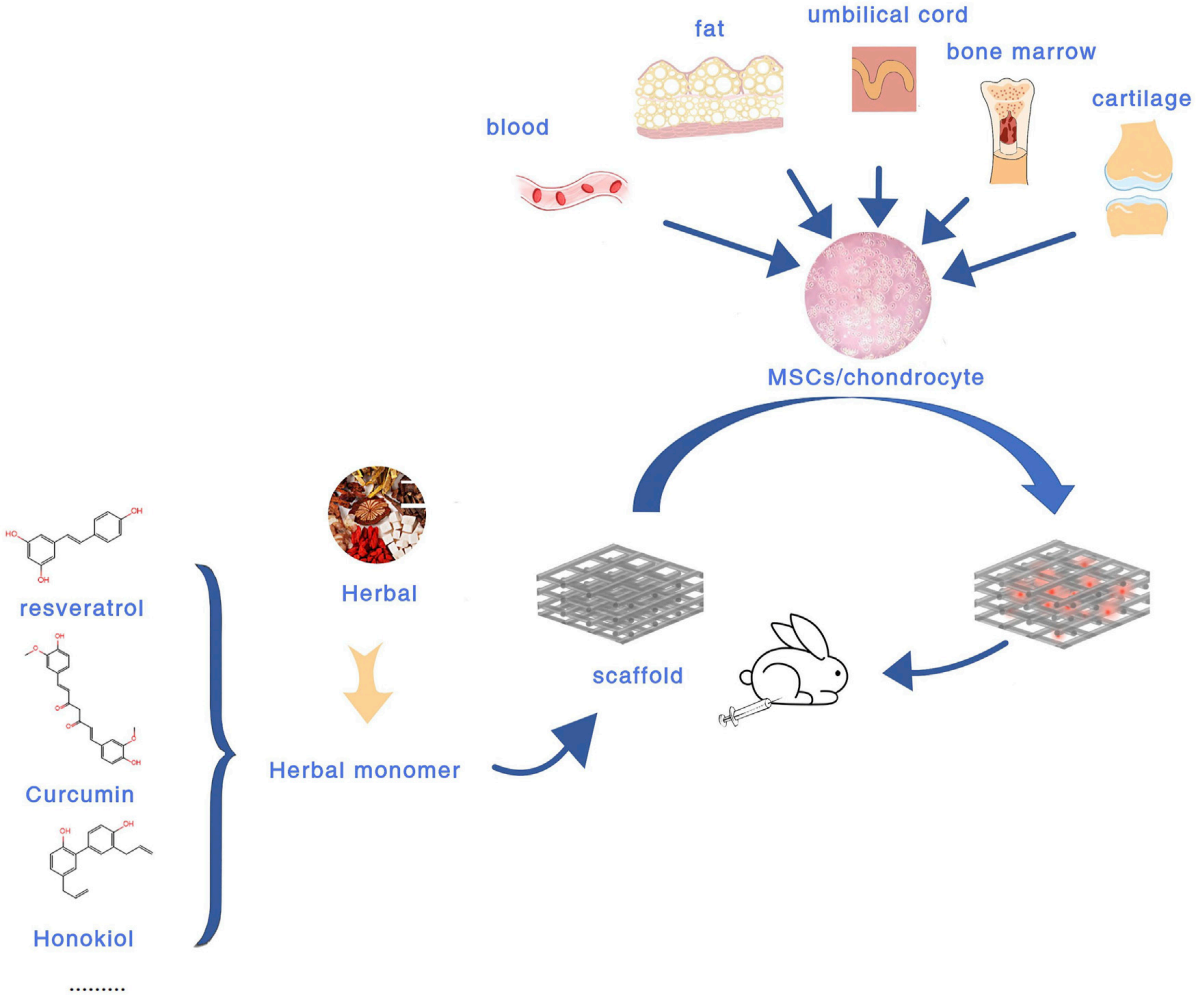
Experimental flow chart of IA injection of BDEs-loaded biomaterial scaffolds.

Compared with the above treatment methods, tissue engineering composed of scaffolds, cells, and favorable growth factors has become the most promising treatment strategy for cartilage repair (Xu et al., 2020). Biodegradable tissue engineering scaffolds have attracted great attention in recent years by virtue of their many advantages (Ming et al., 2018). At the same time, tissue engineering methods are preferred for severe cartilage defects, congenital abnormalities, and elderly patients with limited inherent ability (Lima et al., 2019). Currently, there are various biomaterial scaffolds for cartilage defects, including hydrogels, nanoparticles, microspheres, liposomes, and so on (Lima et al., 2019; Jin, 2020; Xu et al., 2020). In terms of cell selection, the current research shows that chondrocytes and MSCs remain the main sources of seed cells in cartilage repair (Xu et al., 2020). However, the main drawback of using autologous chondrocytes is that when cultured *in vitro*, they are prone to dedifferentiate and develop into a fibroblast phenotype (Vinatier and Guicheux, 2016). (Figure 1 Experimental flow chart of intra-articular injection of BDEs loaded biomaterial scaffolds).

Growth factors play essential regulatory roles in cartilage development (Lecanda et al., 1997; Chen et al., 2020a). The most effective growth factor for inducing cartilage formation is transforming growth factor β (TGF-β) (Makris et al., 2015). However, *in vivo* studies have shown that under continuous stimulation by TGF-β1, adverse reactions such as synovial fibrosis, endochondral osteogenesis, and hypertrophic scarring were observed. On the other hand, growth factors can only release their maximum potential at the injured site and can easily become inactivated under physiological conditions (Chen et al., 2020a). Moreover, the use of growth factors is limited due to their complex extraction process, high cost, and low yield (Wildemann et al., 2007). At present, it is urgent to explore a simple, effective, safe, and cheap bioactive substance to replace growth factors and meet the actual demands of cartilage repair (Kankala et al., 2018; Jin, 2020).

**FIGURE 2:**
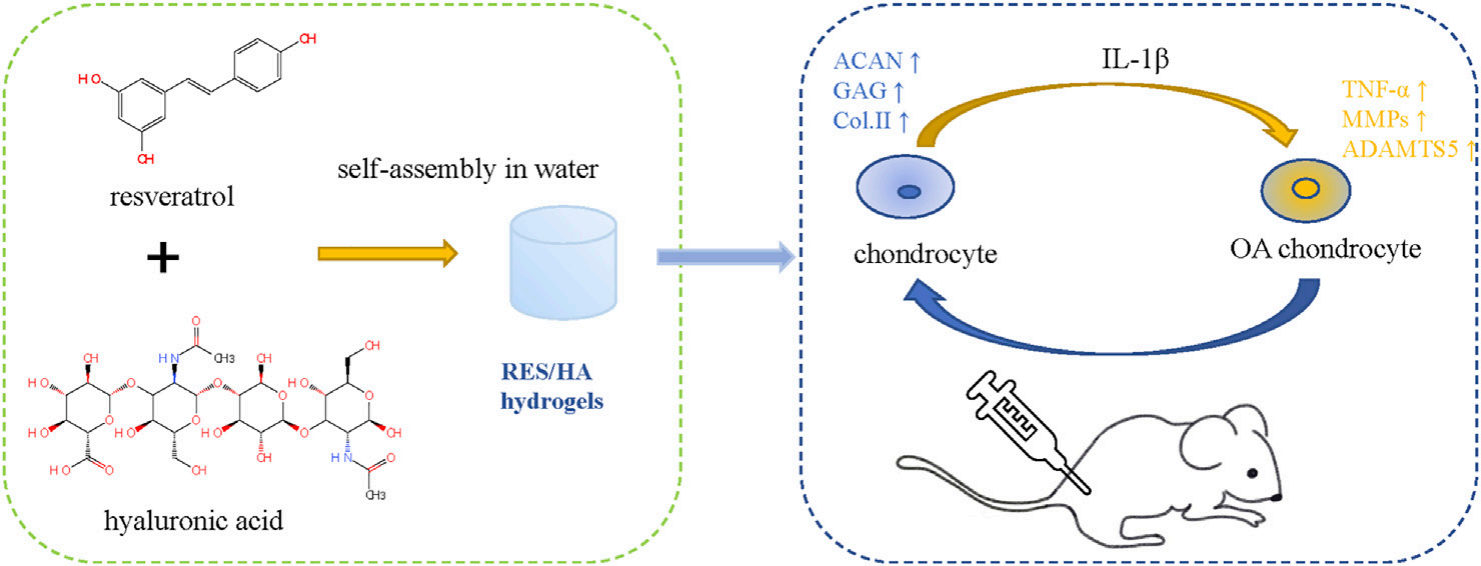
Schematic diagram of chondrocyte regulation of HA-based hydrogel combined with BDEs.

There are many reviews on the repair of cartilage defects. Based on preclinical and clinical studies, Theodoridis et al. (2021) reviewed the research status and limitations of cartilage tissue engineering as a cartilage repair strategy from the aspects of biomaterial selection, cell implantation, scaffold performance and structure, local biological stimulation, etc. Makris et al. (2015) reviewed the current clinical use of articular cartilage surface defect repair technologies and the status of preclinical research on stent-based acellular or chondrocyte implantation technologies. Murphy et al. (2019) mainly discussed the repair technology of meniscus injury and the protective effects of meniscus repair on cartilage. Herbal therapy for OA has been reported in many related studies, and botanical drug extracts (BDEs) combined with scaffolds in the treatment of OA have also been the subject of a large number of studies. Compared to growth factors, BDEs have a wider range of sources, more accessibility, and greater cost-efficiency. Various studies have demonstrated their anti-inflammatory, antioxidant, and cartilage protection properties. Many practices have studied the use of herbs in biotechnology in treating rheumatoid arthritis (RA) and OA (Buhrmann et al., 2020). However, the combination of BDEs with cartilage tissue engineering is rarely used in clinical practice and is mostly done in preclinical research. In this review, we will explain in detail how cartilage tissue engineering materials and BDEs play a role in cartilage repair, as well as the current research status (Figure 2 Schematic diagram of chondrocyte regulation of HA - based hydrogel combined with BDEs).

### 1.3 Botanical Drug Extracts

In the latest guidelines for OA, NSAIDs are recommended as a high-grade evidence-based therapy (Bannuru et al., 2019), but patients are prone to adverse reactions after taking such drugs. In recent years, certain advancements have been made in the treatment of OA with botanical drug remedies. In relevant meta-analyses, it has been found that botanical drug can relieve pain and improve the range of motion in patients with KOA (Cameron and Chrubasik, 2014), and the incidence of adverse reactions is low (Chen et al., 2016a). We searched in the PubMed database for “osteoarthritis,” “arthritis,” “tissue engineering,” “cartilage tissue engineering,” “biomaterials, Chinese herbal medicine,” “scaffolds,” “nanoparticles,” and “hydrogels.” Reviews, conference papers, and studies (those did not include both BDEs and carriers) were excluded. Finally, 31 related articles were selected through reading headlines, abstracts, and full texts. We then classified the BDEs in the literature (including flavonoids, polyphenols, alkaloids, saponins, and others). By summarizing the effect of plant drug extract combined with biomaterial carrier on the repair of OA cartilage injury, its mechanism, and current research status, we provide a theoretical basis for ideas and directions of future research.

## 2 Flavonoids

### 2.1 Quercetin

Quercetin (QUE), an abundant bioflavonoid, is widely distributed in the plant kingdom as a secondary plant metabolite. It is also a common component of the human diet, being found in onions, apples, and so on (Andres et al., 2018). QUE has effective antioxidant, antiproliferative, and free radical scavenging properties (Yeung et al., 2021), as well as being able to alleviate pain (Britti et al., 2017) caused by arthritis (Kanzaki et al., 2012; Wei et al., 2019). A large amount of evidence shows that QUE can delay the occurrence and development of OA (Britti et al., 2017; Feng et al., 2019a; Hu et al., 2019; Ma et al., 2019). In the animal model of OA, QUE can raise the production of superoxide dismutase (SOD), attenuate reactive oxygen species (ROS) levels, reduce inflammation and oxidative stress, improve antioxidant defense, maintain the integrity of joint cartilage ECM, and reduce symptoms and arthritis (Qiu et al., 2018; Wei et al., 2019). QUE can inhibit the expression of chondrocyte matrix degradation protease and inflammatory mediators, reduce cartilage degradation and apoptosis in OA rats, and promote cartilage synthesis (Hu et al., 2019). Siard et al. (2016) showed that QUE significantly reduces the production of inflammatory cytokines by lymphocytes and is even more effective than common NSAIDs. However, due to its low bioavailability of oral administration, studies have reported no significant improvement in the treatment of arthritis with QUE (Bae et al., 2009; Javadi et al., 2014). Mok et al. (2020) injected QUE-loaded polypeptide-based hydrogel, namely methoxy-poly(ethylene glycol)-l-poly(alanine) (mPEG-PA), into the knee joints of OA rats and found that the gradual release of QUE from the mPEG-PA hydrogel could be sustained over 28 days; in addition, the limb idleness index (LII) of the rats was significantly decreased after treatment with QUE (50 μg) hydrogel at 4 and 8 weeks (−0.27, −0.31). The Osteoarthritis Research Society International (OARSI) score shows that the international score of the QUE hydrogel group (50 μg) was significantly lower than that of the control group (*p* < 0.01). These results suggest that the continuous use of QUE hydrogel (50 μg) can relieve OA symptoms and delay the progression of KOA.

### 2.2 Hesperetin

Hesperetin (Hes), a flavonoid in the flavanone class extracted from citrus fruit (Muhammad et al., 2019), can inhibit osteoclast formation and promote the differentiation of osteoblasts, giving it potential for treating bone-related diseases (Ouyang et al., 2019). HES also has antioxidant and anti-inflammatory properties. The antioxidant activity of HES not only is limited to scavenging free radicals but also enhances the defense ability of antioxidant cells through the ERK/Nrf2 signaling pathway (Parhiz et al., 2015). Lin et al. (2020) found the anti-inflammatory effect of HES in OA lies in inhibiting the IL-1β–induced expression of inflammatory factors, downregulating MMP13 and ADAMTS5, and upregulating COL2 and protein aggregation. There has been only one animal study that used HES combined with a cartilage tissue engineering scaffold. Ouyang et al. (2019) loaded HES into nanoparticles (HGdPDW). HGdPDW promoted the expression of ACAN, Sox9, and COL2A1 after IL-1β stimulation for 3 days (*p* < 0.05) but inhibited the expression of MMP13 and COL10A1 (*p* < 0.05), thus reducing apoptosis and inflammatory response. Magnetic resonance imaging (MRI)/ Interactive Video Information System (IVIS) bipeak imaging confirmed that HGdPDW has low cytotoxic and chondrogenic binding ability. HGdPDW treatment increases the articular cartilage thickness of the knee, preserves proteoglycan and collagen, and alleviates the progressive degeneration of articular cartilage *in vivo*. In addition, it can significantly improve the OARSI score of anterior cruciate ligament transection (ACLT) in OA mice (*p* < 0.05).

### 2.3 Icariin

Icariin (ICA), the main component of *Epimedii Folium* (*Epimedium brevicornu* Maxim., *Epimedium sagittatum* (Sieb. et Zucc.) Maxim., *Epimedium pubescens* Maxim., or *Epimedium koreanum* Nakai), is a typical flavonoid compound with anti-inflammatory, anti-oxidative (Zuo et al., 2019), and bone-protective properties (Wang et al., 2020). ICA can treat OA through multiple pathways and targets. It increases the vitality of chondrocytes through reducing the inflammatory damage caused by NF-κB/HIF-2α signaling (Wang et al., 2020); regulates chondrocyte autophagy by mediating the PI3K/Akt/mTOR pathway (Tang et al., 2021) and inhibiting NF-κB; alleviates OA by inhibiting inflammatory cytokines and chondrocyte apoptosis (Liu et al., 2018a; Mi et al., 2018); upregulates the proliferation, osteogenic, and chondrogenic differentiation of bone marrow–derived MSCs (BMSCs); and protects BMSCs from apoptosis through the mitogen-activated protein kinase (MAPK) signaling pathway (Liu et al., 2020a). In a study on ICA and human OA fibroblast-like synoviocytes, it was found that ICA could inhibit the expression of IL-1β, MMP4, and glucose-regulated protein 78 (GRP78), which further reduces the inflammatory response (Pan et al., 2017). Thus, ICA is an effective candidate drug for the treatment of articular cartilage injury, and due to its low price, easy access, and reparative effects on osteochondral defects, it has earned the increasing attention of researchers.

There are two animal studies that used ICA combined with cartilage tissue engineering scaffolds. Kankala et al. (2018) printed porous three-dimensional (3D) sodium alginate (SA) and gel scaffolds with excellent mechanical strength. They then studied the physical characterizations and cytocompatibility of the scaffolds, and the effects of ICA on the growth of chondrocytes. With the extension of the incubation time *in vitro*, the content of GAG in chondrocytes increased gradually, and ICA (10 μg/ml) could significantly accelerate GAG secretion. However, there is no relevant animal model of OA to further verify the reparative effects of ICA combined with stents on OA cartilage defects. Li et al. (2012) used ICA/Type I collagen (ICA/COL) hydrogel encapsulated neonatal rabbit chondrocytes to construct engineering grafts. The results showed that ICA could significantly upregulate the expression of ACAN, Sox9, and COL2 (from 99.7 to 248%), upregulate the synthesis of GAG and COL2, and accelerate the formation of cartilage tissue in the cell hydrogel structure. It could even improve the reparative efficiency of supercritical osteochondral defects in adult rabbit models and promote the fusion of newly formed cartilage and subchondral bone. Thus, ICA is a promising compound for cartilage repair and serves as a substitute for certain growth factors (Table 1).

**TABLE 1:** The effect of flavonoids combined with carriers on OA cartilage repair.

Research (author & year)	BDEs	Carrier	Gene expression		Cartilage repair effects		Other evaluations	Research conclusion
			Inhibition	Promote	Imaging evaluations	Tissue sections		
Ouyang et al. (2019)	Hes	Gd2(CO3)3-PNs	IL-6, TNF-α, NO, iNOS, MMPs, Bax	Col.II, Bcl-2, ACAN, Sox9	Cartilage affinity of synthesized NPs based on MRI/IVIS	Cartilage thickness, caspase-3 expression, and OARSI score	TLR2/NF-κB/Akt signaling pathway	HGdPDW could effectively target cartilage and protect chondrocytes from apoptosis and inflammation
Mok (2018)	Que	MPIP				OARSI score	LIIs (OA-related pain), Krenn score (synovial inflammation)	Sustained delivery of Que (50 μg) could provide symptom relief and also delay the progression of KOA
Kankala et al. (2018)	ICA	SA/gel		GAG			The distribution of chondrocytes on the scaffold surface (by CLSM)	SA/gel composite scaffold addition of ICA further promoted cell proliferation and differentiation
Li et al. (2012)	ICA	Col-CH		Col.I, Col.II, GAG, ACAN, Sox9	Restoration of osteochondral defects by direct observation	Restoration of the osteochondral defects and cartilage thickness	The morphology of chondrocytes encapsulated in hydrogels (by CLSM)	ICA can promote cartilage repair and cartilage tissue formation

CLSM, confocal laser scanning microscopic; EE, Encapsulation efficiency; ILLs, limb idleness index; IVIS, Interactive Video Information System; PNs, polydopamine nanoparticles; MPIP, methoxy-poly(ethylene glycol)-l-poly(alanine); Col-CH, collagen type I cell–hydrogel.

## 3 Polyphenols

### 3.1 Resveratrol

Resveratrol (RES) is a phytochemical found in grape and *Reynoutria japonica* Houtt. which acts on several cellular signaling pathways and has anti-inflammatory, antioxidant, and antiaging properties (Limagne et al., 2016; Wang et al., 2016; Oz et al., 2019; Yeung et al., 2019; Yuce et al., 2021). Elmali et al. (2007) showed that the IA injection of RES could mitigate synovitis cell infiltration and cartilage damage through the histopathological evaluation of the rabbit arthritis model. RES can prevent the degradation of proteoglycan and ACAN in cartilage tissue induced by advanced glycation end products (AGEs) (Liu et al., 2010); upregulate the expression of COL2; downregulate the expression of inducible NO synthase (iNOS) and MMP13 (Li et al., 2015); reduce the accumulation of ROS and hypoxia inducible factor-α (HIF-1α); and inhibit the MAPK signaling pathway to prevent inflammation and degeneration of the joints (Yang et al., 2018). RES can also effectively reverse IL-1β–induced catabolic and inflammatory responses (Gu et al., 2017) and significantly prevent OA cartilage damage by activating sirtuin 1 (SIRT1) to inhibit the expression of HIF-2α and catabolic factors (Li et al., 2015; Wang et al., 2016; Abed et al., 2017). In addition, RES has a protective effect on subchondral bone, which is manifested in the promotion of the Wnt/β-catenin and ERK1/2 signaling pathways to boost mesenchymal cell differentiation into osteoblasts (Abed et al., 2017).

Qin et al. (2017) have shown that the IA injection of RES promotes chondrocyte autophagy (*via* regulating the HIF-1α–dependent MAPK/mTOR signaling pathway) and delays cartilage degeneration induced by medial meniscal instability surgery. Dietary RES supplementation can reduce paw edema and erythema in collagen-induced arthritis–OA (CIA-OA) model rats and mitigate the invasion of inflammatory cells and cartilage degeneration around the joints (Oz et al., 2019). The intraperitoneal injection of RES (5 or 10 mg/kg) in monosodium iodoacetate–induced arthritis–OA (MIA-OA) rats significantly reduced mechanical, thermal, and cold hyperalgesia and increased vertical and horizontal movements (Wang et al., 2016). At the same time, a clinical study showed that treatment with RES improved WOMAC scores (pain, stiffness, and body function) (Hussain et al., 2018). These results suggest that RES may be a potential analgesic agent that can reduce pain and discomfort in KOA patients and improve their general condition and life quality. The anti-inflammatory effects of RES have been studied in both humans and animals. Due to the weak oral bioavailability of RES, we hope to develop different administration methods in order to obtain good results in clinical treatment (Kotha et al., 2006).

There are six studies that used RES combined with cartilage tissue engineering scaffolds to treat OA. Kann et al. (2016) encapsulated RES and curcumin (CUR) in lipid-core nanocapsules and studied their interaction with the human primary chondrocytes. Due to the different solubilities of CUR and RES, the lower solubility accompanied faster release from the nanocapsules. The combination of the two can achieve the effect of rapid initial treatment (by RES) and prolonged treatment (by CUR). By virtue of its rapid release, RES can quickly clear ROS produced in OA rats induced by sodium nitroprusside (SNP) and reduce NO expression, thereby protecting the chondrocytes. Coradini et al. (2015) also studied the synergistic effects of RES and CUR using intraperitoneal injections instead of IA injections. RES and CUR were co-encapsulated in lipid core nano-capsules and intraperitoneally injected into Complete Freund’s adjuvant (CFA)–induced arthritis rats. The results showed that co-encapsulated polyphenols were safe and nontoxic when injected *in vivo* and could significantly reduce foot swelling in arthritic rats (*p* < 0.05), with an amplitude of 37–55%. In addition, co-encapsulated polyphenols alleviated cartilage damage by significantly reducing synovial membrane fibrosis and cartilage and bone loss (*p* < 0.05). Ming et al. (2018) used the microfluidic technique to select the optimum concentration of chondrocytes cultured by RES and then studied the reparative effects of RES and drug-loaded polylactic acid (PLA)/gelatin nano-scaffolds on cartilage defects. They reported that the best concentration of RES was 114.281 mol/L. Nanofiber scaffolds have good elasticity, mechanical strength, and compression recovery ability and can play a supporting role in the repair of cartilage defects. The results of animal experiments also showed that the PLA/gelatin 3D nano-scaffold could promote the repair of cartilage defects. RES (114.281 mol/L) loaded inside the scaffold could greatly promote the differentiation of chondrocytes and formation of cartilage, and maintain the normal structure of the new cartilage, with good cartilage defect reparative effects and improved OA treatment effects. Qin et al. (2020) prepared RES-loaded silica aerogel (RSA) using the sol–gel method. In simulated gastric juice (pH = 2.0) and a phosphate buffer (pH = 7.4) at 37°C, the release of RSA lasted for more than 6 h, and the release amount reached 90 and 80%, respectively. Preliminary *in vitro* toxicity tests showed that RSA had good biocompatibility and stability. Combined with the anti-inflammatory effects of RES, it shows great potential for treating OA. Sheu et al. (2013) prepared an oxidized HA (Oxi-HA)/RES hydrogel, which showed good biocompatibility. The Oxi-HA/Res hydrogel could upregulate the gene expression of COL2, aggrecan, and Sox9; promote ECM synthesis; downregulate the gene expression of IL-1B, MMP1, MMP3, and MMP13; and reduce inflammation and injury induced by lipopolysaccharides. The Oxi-HA/Res hydrogel may be a potential chondrocyte carrier for the treatment of cartilage defects. However, this study only carried out *in vitro* experiments, so further *in vivo* research is necessary to determine possible clinical application in the future. Kamel et al. (2019) prepared a mixed micellar system using poloxamer (P188:P407 = 2:1), then subjected it to *in vitro* and *in vivo* evaluation, and compared the curative effects of MM3, PLA-coated MM3, and drug suspension. PLA-coated MM3 had the best ability to eliminate knee edema, and MM3 had the best ability to remove TNF-α. The addition of PLA coating can increase drug retention and reduce drug clearance from the synovial fluid. Histological studies have shown that articular and synovial structures can be restored through IA injection of drug-loaded micellar nano-systems. In addition, it can also reduce the symptoms of joint swelling in OA rats (Table 2).

**TABLE 2:** The effect of RES combined with carriers on OA cartilage repair.

Research (author & year)	BDEs	Carrier	Gene expression		Cartilage repair effects		Other evaluations	Research conclusion
			Inhibition	Promote	Imaging evaluations	Tissue sections		
Kann et al. (2016)	CUR and RES	PC-NPs	NO		No mention	No mention	Microscopy; cell viability testing	Polyphenols combined with nanocapsules can significantly reduce the level of NO, protect joint cells, and prevent apoptosis
Coradini et al. (2015)	CUR and RES	Lipid-core nanocapsules			No mention	Significantly attenuation of fibrosis in the synovial membrane, cartilage, and bone loss		
Ming et al. (2018)	RES	PLA-gel			Significant articular cartilage repair effect were assessed by general observation and microCT	A smooth and thick cartilage surface and a clear structure were observed by hematoxylin and eosin staining, Safranin-O Fast Green staining, Alcian blue staining, and toluene staining		PLA/gelatin 3D nano scaffolds loaded with RES can greatly promote the formation of cartilage
Qin et al. (2020)	RES	SiA			No mention	No mention	Surface morphology analysis, FTIR analysis, Raman analysis	RSA has biocompatibility and stability; Combined with the anti-inflammatory effect of RES, it shows a good potential in the treatment of OA
Sheu et al. (2013)	RES	HA	IL-1β, MMPs, Col.I	Col.II, ACAN, Sox9	No mention	No mention	MTT, FTIR, and TNBS assays	Oxi-HA/RES hydrogel is biocompatible with chondrocytes, allowing ECM synthesis and reducing LPS-induced inflammation and injury
Kamel et al. (2019)	RES	PLA-P	TNF-α					RES-loaded mixed micellar nanosystems reduce the severity of cartilage injury and synovitis

EE, Encapsulation efficiency; FTIR, Fourier transform infrared; PLA-P, PLA-coated poloxamer; HA, hyaluronic acid hydrogel; SiA, silica aerogel; PLA-gel, PLA/gelatine nano-scaffold; PC-NPs, poly(ε-caprolactone) nanocarriers; MTT, MTT assay; TNBS, trinitrobenzene sulfonate assay.

### 3.2 Curcumin

CUR, a bioactive polyphenolic, is a natural BDEs which is mainly extracted from the rhizome of turmeric (*Curcuma longa L.*) (Feng et al., 2019b). CUR has significant antioxidant and free radical scavenging effects and is traditionally considered to possess anti-inflammatory, anticancer, antioxidant, antiangiogenesis, and anti-radiation properties (Aggarwal et al., 2013; Tasneem et al., 2019; Hasanzadeh et al., 2020).

Numerous previous studies have shown that CUR can inhibit chondrocyte apoptosis (Zhang et al., 2016; Henrotin et al., 2019). Its molecular mechanisms are not only associated with the inhibition of cell proliferation and metastasis but also associated with the downregulation of various factors such as TNF-α, IL-1β, and protease levels (Khayyal et al., 2018; Wang et al., 2019). Extensive research has revealed that CUR can exhibit anti-inflammatory effects on OA *via* suppressing the NF-κB signaling pathway, IL‐1β, IL‐6, IL‐18, iNOS, prostaglandin E2 (PGE_2_), and cyclooxygenase-2 (COX-2) (Chin, 2016; Zhang et al., 2016; Qiu et al., 2020). Chondrocyte apoptosis in OA is related to oxidative and endoplasmic reticulum (ER) stress (Feng et al., 2019b). Feng et al. (2019b) demonstrated that CUR protected rat chondrocytes from apoptosis by promoting SIRT1 expression and inhibiting ER stress and its related PERK-eIF2α-ATF4-CHOP signaling pathway.

Yang et al. (2007) compared the routes of oral and intravenous administration of CUR and found that due to the very low oral bioavailability of CUR, its maximum plasma concentration (500 mg/kg) is much lower than that of intravenous injection (10 mg/kg) (0.06 vs. 0.36 μg), and so a very high dose (>10 g/day) is required for oral administration to reach the plasma concentration recommended in the preclinical study (Belcaro et al., 2010; Gupte et al., 2019). A clinical study showed that CUR and low-dose ibuprofen had the same effect in reducing OA pain (Kuptniratsaikul et al., 2014; Gupte et al., 2019). A meta-analysis showed that the use of CUR as a dietary supplement was better than glucosamine (GA) and chondroitin in improving OA in the knee joints, hip joints, and hands in the short term (Liu et al., 2018b). Other studies have also shown that CUR can reduce OA pain and improve joint function while reducing the risk of adverse events (Panahi et al., 2014; Bannuru et al., 2018; Zeng et al., 2021) and can serve to decrease the use of NSAIDs which has more adverse effects (Grover and Samson, 2016; Wang et al., 2021a). At present, there are many methods, such as nanoparticles, liposomes, micelles, and phospholipid complexes, which can extend drug release time and increase permeability and resistance to clearance (Anand et al., 2007; Wang et al., 2019).

There are five studies that used CUR combined with a cartilage tissue engineering scaffold to treat OA. Kang et al. (2020) added CUR to the hydrophobic main chain of poly(β-amino ester) (PAE) to prepare anti-inflammatory polymeric prodrug of CUR (ACP) and found that amphiphilic ACP dissociated under acidic conditions. The potential of ACP micelles as a targeted therapeutic agent for inflammatory disease was investigated using an MIA-OA rat model due to the pathological characteristics of OA joints, such as low pH (6.6–7.1) and high ROS levels. In an acidic environment, the CUR release rate of ACP was very fast, 95% in 7 days, showing that it can play a role in accelerated drug treatment. ACP could significantly scavenge H_2_O_2_ and had no cytotoxicity when the concentration was lower than 100 μg/ml. ACP could also significantly inhibit the levels of TNF-α and IL-1β, with a stronger effect than that of free CUR. After treatment with ACP micelles, the articular surface of MIA-OA rats was smooth, the cartilage structure was intact, and the expression of proteoglycan, ACAN, and collagen protein was strong. The results showed that ACP micelles had strong anti-inflammatory and anti-arthritis properties. Yeh et al. (2015) used soybean phosphatidylcholines as a liposome formulation and compared the particle size, encapsulation efficiency, liposome stability, and cellular uptake of CUR/bisdemethoxycurcumin (BDMC)–loaded liposomes. The results showed that the encapsulation rates of CUR and BDMC in the liposomes were 69.5 and 71.4% respectively, and the particle size of the liposomes was stable after formation. Both liposomes inhibited macrophage inflammation and osteoclast differential activity. Compared with free drugs (such as CUR and BDMC), CUR-like liposomes (CUR-LIP) showed less cytotoxicity and a higher drug uptake rate. CUR-LIP can inhibit the proliferation of osteoclasts and maintain the differentiation function of osteoblasts by increasing the ratio of osteoprotectin (OPG)/NF-κB ligand receptor activator (RANKL), downregulating MMP3 and COX-2 induced by IL-1β. Therefore, CUR liposomes may delay the progression of OA.

Crivelli et al. (2019) prepared silk fibroin nanoparticles (SFNs) using the desolvation method, then tested empty and drug-loaded [with CUR or celecoxib (CXB)] nanoparticles for their ROS scavenging activity, hemolysis, cytotoxicity, and anti-inflammatory effects in an OA *in vitro* model. The results indicated that CUR-SFNs exhibited a synergistic antioxidant effect. SFNs encapsulation could reduce the cytotoxicity of free drugs. CXB, a selective COX-2 inhibitor, is the first choice in the treatment of OA pain, but the long-term high-dosage use of CXB may cause serious cardiotoxicity and renal complications. On the contrary, CUR can reduce OA-related inflammation without obvious side effects. When free CUR was added to SFNs, its antioxidant activity increased from 80 to 90%, while CBX showed no significant difference. Based on these findings, it can be speculated that the combination of SFNs and CUR has synergistic antioxidant effects. At high concentrations, the anti-inflammatory effects of SFNs/CUR (400 μg/ml) are greater than those of SFNs/CXB (800 μg/ml). Therefore, we believe that SFNs/CUR is more suitable for the treatment of OA than SFNs/CXB. Of course, more *in vivo* and preclinical studies are needed to verify this view.

Wang et al. (2018) prepared HA/chitosan nanoparticles (cNP) for the delivery of CUR to investigate their effect on OA treatment. The results showed that the optimal drug loading of HA/cNP for CUR was 38.44%, which showed a good sustained release effect. CUR delivered by HA/cNP inhibiting the inflammation and chondrocyte apoptosis of OA may be mediated by inhibiting the NF-κB pathway and expression of MMP1 and MMP13, and increasing the expression of COL2. Ratanavaraporn et al. (2017) prepared gelatin/silk fibroin microspheres loaded with CUR for use in the anti-inflammatory treatment of MIA-OA rats. The results showed that the encapsulation efficiency of CUR by gelatin/silk fibroin (30/70) microspheres reached 59%, the encapsulation amount of CUR microspheres was about 2 μg/mg. CUR-coated gelatin/silk fibroin (30/70) microspheres reduced serum IL-6 levels and delayed the degeneration of joint and synovial tissue cells. These results indicate that the CUR-sustained release system can be used for the local anti-inflammatory treatment of OA with reduced trauma.

### 3.3 Epigallocatechin-3-Gallate

Epigallocatechin-3-gallate (EGCG), a major extract of green tea [*Camellia sinensis* (L.) Kuntze], accounts for approximately 40–60% of green tea polyphenols (Zheng et al., 2019). EGCG can inhibit the activation of p38 MAPK and c-Jun N-terminal kinase (JNK) (Singh et al., 2003) and significantly reduce the levels of TNF-α and MMP13 in human chondrocytes stimulated by AGEs (Rasheed et al., 2016). With both anti-inflammatory and antioxidant properties (Akhtar and Haqqi, 2011), EGCG inhibits COX-2 and PGE_2_ production *via* the upregulation of hsa-miR-199a-3p expression (Ahmed et al., 2002; Ownby et al., 2014; Jhang et al., 2016), inhibits IL-1β–induced ADAMTS5 expression (Rasheed et al., 2016), and cartilage and proteoglycan degradation (Heinecke et al., 2010). Mice treated with EGCG showed OA-related pain relief (Leong et al., 2014), indicating that EGCG can not only delay the progression of OA but also relieve its symptoms. Natarajan et al. (2015) verified the cartilage protective effects of EGCG through the IA injection of polyphenols, but it requires repeated injections, which is not popular in clinical medicine.

There are two animal studies that used EGCG combined with cartilage tissue engineering scaffolds. GA, an amino monosaccharide, is an important component of polyglucosamine which is found mainly in the cartilage matrix and synovial fluid. Its protective effects on cartilage are mainly inhibiting proteoglycan degradation, stimulating proteoglycan synthesis, inhibiting the activation of inflammatory cells, activating chondrocytes and synovial cells, and restoring joint function. Zheng et al. (2019) studied the effect mechanism of an EGCG-GA mixture and EGCG-GA-casein protein nanoparticles (EGCG-NPs) in OA rats. The results showed that EGCG, as a plant polyphenol, could significantly promote the effects of GA; nanoparticles (NPs) could significantly improve the stability of EGCG; and EGCG combined with NPs could inhibit TNF-α, IL-1β, IL-6, and IL-8 expression in OA rats, inhibit synovial hyperplasia and inflammatory cell infiltration, and effectively alleviate inflammation. It could also improve erythema and swelling around the joints in OA rats. The other study combined EGCG with HA and gelatin to create a composite hydrogel in order to explore the anti-inflammatory and cartilage protective effects of an EGCG-loaded composite hydrogel in the treatment of OA *in vivo* and *in vitro* (Jin et al., 2020b). After adding EGCG to the hydrogel, the cytotoxicity of EGCG was eliminated and it showed good biodegradability. Moreover, the expression of IL-1β, TNF-α, MMP13, and ADAMTS5 were significantly downregulated. Under *in vitro* experiment conditions, EGCG could promote the growth and differentiation of chondrocytes. After 4 weeks of injection into the joints of OA rats, no significant differences were observed in the surface or thickness of the cartilage between the injected rats and control rats. Therefore, we believe that EGCG combined with material scaffolds will be a promising tissue engineering strategy for repairing cartilage defects in OA.

### 3.4 Honokiol

Honokiol (HON) is a phenolic compound extracted from *Magnolia officinalis* Rehder & E.H.Wilson (Kim et al., 2010). *In vitro* studies of macrophages and neutrophils showed that HON had anti-inflammatory effects (Munroe et al., 2007). Other previous studies showed that HON may have a cartilage protective effect (Chen et al., 2014; Wu et al., 2017a). In the CIA-OA mice, the oral administration of magnolol and HON could block the production of IL-17, MMPs, RANKL, and NO in inflammatory joints and reduce serum TNF-α and IL-1β to inhibit inflammation, cartilage degeneration, and bone erosion (Kim et al., 2010) with no liver or kidney damage. However, its oral bioavailability is low, and only 1% can be absorbed. Moreover, HON can inhibit the levels of PGE_2_, NO, TGF-β1, and IL-6, the protein expression of COX-2 and iNOS, and the phosphorylation of Akt, IκB-α, and NF-kB, upregulated by IF-1β (Li et al., 2011; Chen et al., 2014). The results of Park et al. (2017) showed that HON could inhibit the osteoclastic effect mediated by RANKL. In addition, HON promotes the chondrogenesis of human umbilical cord MSCs and inhibits inflammation by blocking the NF-κB pathway (Wu et al., 2017a). There is only one animal study that uses HON combined with a cartilage tissue engineering scaffold. Zhu et al. (2020) showed that HON significantly inhibited the release of pro-inflammatory cytokines TNF-α, IL-1B, and IL-6 stimulated by lipopolysaccharide (LPS). *In vivo*, a scaffold containing HON was implanted into the cartilage defect area. After 8 weeks, the defect area was filled with COL2-positive cartilage tissue with an intact tide line structure, and the cartilage had become hard and smooth. Meanwhile, the ratio of the bone volume to tissue volume (BV:TV) was significantly increased. These results show that HON combined with a scaffold can promote the regeneration of hyaline cartilage and bone tissue in osteochondral defects (Table 3).

**TABLE 3:** The effect of polyphenol active ingredients combined with carriers on OA cartilage repair.

Research (author & year)	BDEs	Carrier	Gene expression		Cartilage repair effects		Other evaluations	Research conclusion
			Inhibition	Promote	Imaging evaluations	Tissue sections		
Kang et al. (2020)	CUR	PEG	IL-1β, TNF-α	Col.II, ACAN, Sox9	No mention	Smooth surface with structural integrity of cartilage, along with strong expression of proteoglycan, ACAN, and collagen, was observed by H&E, Masson's trichrome, Safranin-O, and ACAN staining		ACP micelles inhibit TNF-α and IL-1 β, significant protection of joint structure from arthritis
Yeh et al. (2015)	CUR	SPC-liposome	TRAP, cathepsin K, NO, MMPs, COX-2	OPG/RANKL	No mention	No mention	OPG/RANKL signaling pathway	CUR-loaded liposomes can inhibit macrophage inflammation and osteoclast differentiation, which may slow down the progression of OA
Crivelli et al. (2019)	CUR	SNPs	IL-6, RANTES, ROS, NO		No mention	No mention	FTIR, DSC, TGA, SEM	CUR and SFNs showed synergistic antioxidant effect
Wang et al. (2019)	CUR	HA/cNP	MMPs, NF-κB	Col.II	Improved articular surface injury in OA rats by general observation *via* a microscope	The knee joint surface was smooth, and the cells were regularly arranged in OA rats by H&E staining, toluidine blue staining, Safranin-O Fast Green staining	UV assay; flow cytometry; western blot analysis	HA/cNP and CUR may suppress inflammation and chondrocyte apoptosis in KOA *via* repression of the NF-κB pathway
Ratanavaraporn et al. (2017)	CUR	Gel/SMs	IL-6		Sign of OA was not observed in the treatment group *via* X-ray	Histologic and histochemical grading of articular joint and synovial tissue change of OA rats treated with CUR-loaded gel/SMs was significantly better than in other groups	Radiographic, histological examination	CUR gel/SMs have potential anti-inflammatory effect on OA joint in rats
Jin. (2020)	EGCG	HA/gel	IL-1β, TNF-α, ADAMTS5, MMPs	Col.II, ACAN, Sox9	No mention	In 5% HTG-E group, cartilage surface and thickness were completely intact, showing no signs of wear and tear		HTG hydrogel can promote the accumulation of ECM, and it has anti-inflammatory and cartilage protective ability after loading EGCG
Zheng et al. (2019)	EGCG	EGC-NPs	IL-1β, IL-6, TNF-α		The therapeutic effect of the EGC-NPs was significantly better than that of the EGCG-GA mixture and comparable to the antiarthritic effect of celecoxib by a radiographic evaluation and scoring system	Combined with EGCG, GA can effectively promote its antiarthritic effects		The anti-inflammatory effect of EGC-NPs was significantly higher than that of the EGCG-GA mixture
Zhu et al. (2020)	HON	ECM/PGDH	IL-1β, IL-6, TNF-α	Col.I, Col.II, BV:TV, Tb.Th	In the group in which the defect was repaired with PEGDA/ECM/HON scaffold, the surfaces of the defect were smooth and the defect region displayed an intact tideline structure *via* micro-CT	The defects in the PEGDA/ECM/HON group were mostly filled with cartilage tissue positive for COL2, indicating regeneration of hyaline cartilage-like tissue		Scaffolds combined with HON promoted the regeneration of hyaline cartilage and subchondral bone

EE, Encapsulation efficiency; CFA, Complete Freund’s adjuvant; H&E, hematoxylin and eosin; SNP, sodium nitroprusside; FTIR, Fourier transform infrared; Tb.Th, trabecular thickness; HA/cNP, HA/chitosan nanoparticles; gel/SMs, gelatin/Thai silk fibroin microspheres; HA/gel, HA/gelatin hybrid hydrogel; ECM/PGDH, 3D-printed ECM/polyethylene glycol diacrylate hydrogel; SNPs, silk fibroin nanoparticles; PEG, poly(ethylene glycol); HA, hyaluronic acid hydrogel; PC-NPs, poly(ε-caprolactone) nanocarriers; UV, ultraviolet; DSC, Differential scanning calorimetry; TGA/DSC 1, Simultaneous thermogravimetric analysis; SEM, scanning electron microscopy; HTG, HA/gelatin; SPC, Soybean phosphatidylcholine; TRAP, tartrate-resistant acid phosphatase; HTG-E, EGCG-loaded HA/gelatin; EGC-NPs, EGCG-GA-Casein Nanoparticles.

## 4 Alkaloids

### 4.1 Brucine

Brucine (BRU) is a major effective component of *Strychnos nux-vomica* L. (Wu et al., 2017b), with high toxicity (Liu et al., 2015a; Lu et al., 2020). *S. nux-vomica* L. has a long history of clinical application in herbal medicine, especially in the treatment of RA and OA. BRU has anti-inflammatory and analgesic effects (Yin et al., 2003) and can significantly inhibit LPS-induced PGE_2_ production (Liu et al., 2015a; Lu et al., 2020). BRU can effectively promote chondrocyte regeneration and repair cartilage damage caused by OA. Although BRU is highly effective in the treatment of OA, its potential use is severely limited due to its high toxicity; with its good lipid solubility, it can easily penetrate the blood–brain barrier and cause serious toxicity to the central nervous system if distributed in the brain (Gao et al., 2021). Its oral administration can also cause gastrointestinal irritation and systemic toxicity (Liu et al., 2015a; Lu et al., 2020). Until now, BRU has not been used clinically or studied in clinical trials. Therefore, new drug delivery systems are urgently needed to reduce the side effects (Liu et al., 2015a; Lu et al., 2020). There is only one animal study that used BRU combined with a cartilage tissue engineering scaffold. To determine the feasibility of IA injection, Chen et al. (2012) evaluated a BRU-loaded microsphere/thermally responsive hydrogel (BMH) combination system in such aspects as drug release, pharmacodynamics, and biocompatibility. The results showed that the entrapment rate of this system was 98.60% w/w with an average particle size of 0.9–4.5 μm. The sustained release cycle of BMH was 7 days. They established a rabbit OA model through the IA injection of collagenase and studied the therapeutic effects of this strategy on OA by IA injection. Histological assessment showed that BMH reduced the number of fibroblasts and improved synovial integrity during cartilage defect repair, and the pharmacodynamic results revealed that BMH could protect OA joints from degradation by suppressing TNF-α and IL-1β levels.

### 4.2 Sinomenium

Sinomenium (SIN) is a BDE extracted and purified from the plant *Sinomenium acutum* (Thunb.) Rehder & E.H.Wilson (Chen et al., 2016b), which has anti-rheumatism, anti-inflammatory, and anti-pain effects (Wu et al., 2019a; Jiang et al., 2020). Clinically, SIN is widely used to treat patients with RA (Zhao et al., 2012). By activating the Nrf2/HO-1 signaling pathway and inhibiting NF-κB activity, SIN can reduce the protein levels of ADAMTS5 and MMPs in rats (Wu et al., 2019b), inhibit inflammation and ECM degradation, and play a role in cartilage protection (Wu et al., 2019a). In addition, SIN downregulates iNOS, COX-2, NO, PGE_2_, TNF-α, and IL-6 induced by IL-1β (Wu et al., 2019a), suggesting that it protects chondrocytes by promoting autophagy and preventing cartilage degradation, thereby reducing the clinical symptoms of arthritis. However, few studies have evaluated the availability of SIN in the treatment of OA. There is only one animal study that used SIN combined with a cartilage tissue engineering scaffold. Chen et al. (2016b) evaluated the therapeutic effects of SIN coated with chitosan microspheres (CM-SIN) on OA by IA injection. Both *in vivo* and *in vitro* studies showed that SIN could partially induce autophagy to inhibit the degradation of the cartilage matrix induced by IL-1β. SIN downregulates the mRNA expression of cartilage degradation markers MMP13 and ADAMTS5 and upregulates the expression of ECM components COL2A1 and ACAN. After the IA injection of CM-SIN for 4 and 8 weeks, the OARSI score of OA mice was significantly reduced (*p* < 0.01), cartilage degeneration was improved, and OA progression was delayed.

### 4.3 Berberine

Berberine (BBR) is an active component isolated from *Coptis chinensis* Franch.(“Huanglian” in Chinese) (Neag et al., 2018). BBR has obvious immunosuppressive, anticancer (Mishra et al., 2019), and anti-inflammatory effects (Hu et al., 2011; Zhou et al., 2015a; Belwal et al., 2020). It has been shown that BBR can activate the Wnt/β-catenin signaling pathway in OA rats (Zhou et al., 2016a). Wnt signaling molecules play an important role in the process of osteogenesis (Wong et al., 2020), so it can be speculated that BBR can upregulate the Wnt signaling pathway and promote the reconstruction of the subchondral bone. BBR has also been reported to enhance autophagy levels in chondrocytes (Chen et al., 2018). BBR significantly inhibits IL-1β–induced inflammation by inhibiting the NF-κB signaling pathway in human OA chondrocytes (Zhou et al., 2016b; Lu et al., 2019) and significantly inhibits the IL-1β–induced expression of MMP3, MMP13 (Moon et al., 2011; Belwal et al., 2020), iNOS, and COX-2, and the production of NO and E2. In addition, BBR inhibits apoptosis and promotes the proliferation of SNP-stimulated chondrocytes in rats and OA rats (Zhou et al., 2015b; Zhou et al., 2017). BBR also promotes articular chondrocyte survival and stroma formation by activating Akt signaling (Zhao et al., 2014; Liu et al., 2015b). However, BBR has poor water solubility, low bioavailability, and a short biological half-life, so it is necessary to design a continuous delivery system to improve its utilization.

There are three animal studies that used BBR combined with cartilage tissue engineering scaffolds. Chen et al. (2018) investigated the effects of a combined BBR and sodium hyaluronate/SA (HA/SA) scaffold on cartilage repair. *In vitro*, the scaffold sizes ranged from 100 to 200 μm, and all BBR was released within 72 h. HA/SA-IPN scaffolds combined with BBR can activate the Wnt/β-catenin signaling pathway to promote the osteogenesis differentiation of BMSCs and increase the bone volume to tissue volume ratio (BV/TV). Meanwhile, the cartilage defect surface of the BBR and scaffold group was smooth and filled with thicker hyaline cartilage–like tissue. *In vivo*, BBR significantly increased the level of autophagy marker LC3, downregulated MMP13 and ADAMTS5, and upregulated COL2A1 and ACAN. These results suggest that BBR inhibits the degradation of the cartilage matrix by enhancing autophagy and promoting cartilage regeneration. We believe that this system can regenerate not only cartilage but also subchondral bone simultaneously, which is a promising strategy for osteochondral defect repair. Zhou et al. (2015a) successfully synthesized CNs for the sustained release of BBR using the ionic cross-linking method and established an OA rat model by ACLT combined with medial meniscal resection (ACLT + MMx). *In vitro*, the CNs could continuously release BBR, with good stability, uniform morphology and structure, good particle size and appropriate Zeta potential, and encapsulate a large amount of BBR. In the ACLT + MMx rat model, BBR significantly downregulated the mRNA expression of caspase-3 and Bax and upregulated the mRNA expression of Bcl-2. The Mankin score was used for histopathological scoring, and the results showed that the BBR-CNs group was significantly better than the model group in structural changes, chondrocyte changes, tide mark, and safranin staining (*p* < 0.001), and the cartilage damage of OA rats was reversed. These results suggest that the IA administration of BBR-CNs may be an effective treatment for OA. Zhou et al. (2017) successfully synthesized a new type of BBR-loaded chitosan microspheres (CMs) with an encapsulation efficiency of 100.8 ± 2.7 mg/g. CMs loaded with BBR significantly inhibited the protein expression levels of caspase-3, disintegrin, ADAMTS5, and MMP13 induced by SNP, which confirmed its antiapoptotic activity. However, they did not conduct further animal model tests to verify the system’s effectiveness *in vivo*.

### 4.4 Colchicine

Colchicine (CLC) is a water-soluble alkaloid extracted from *Colchicum autumnale* L. (Mohamed et al., 2020) and the first therapy of choice in gout treatment (Letter, 1974). Due to its anti-inflammatory and anti-fibrosis properties (Leung et al., 2018), CLC is used not only in the treatment of arthritis but also as a remedy to treat numerous skin conditions (Sullivan et al., 1998), Familial Mediterranean fever (Knieper et al., 2017), Behcet's disease (Nava et al., 2014), cirrhosis (Kershenobich et al., 1988; Gong and Gluud, 2004; Rambaldi and Gluud, 2005), and certain carcinomas (Mohamed et al., 2020). Clinical studies have shown that CLC can significantly improve symptoms in KOA patients (Das et al., 2002). However, a study by Leung et al. (2018) showed that CLC (0.5 mg, twice daily, orally, 16 weeks) did not reduce KOA symptoms but reduced the inflammatory and high bone turnover biomarkers associated with OA severity and risk of progression. The oral administration of CLC has certain limitations due to its extensive first-pass effects, poor bioavailability, and severe gastrointestinal side effects (Abdulbaqi et al., 2018). At the same time, the intravenous administration of CLC can cause serious or even fatal effects such as cell loss, tissue necrosis, and intravascular coagulation transmission (Evans et al., 1996; Abdulbaqi et al., 2018), and its intravenous administration is prohibited. In addition, the high water and poor skin permeability of CLC pose challenges for its transdermal delivery (Mohamed et al., 2020). Most importantly, the chronic and progressive nature of OA requires frequent administration. Thus, in order to ensure better safety and effectiveness, it is urgent to find an alternative administration route for CLC (Marwah et al., 2016).

There is only one animal study that used CLC combined with a cartilage tissue engineering scaffold. Mohamed et al. (2020) developed novel CLC transdermal delivery systems to conquer such obstacles. Compared with the drug suspension liquid, the patch increased the drug flux and penetration level for more than 24 h. The therapeutic effects of the CLC patch on the MIA-OA rat model showed that CLC improved exercise ability, increased the blood glutathione level, and significantly reduced the malondialdehyde, NO, TNF-α, and COX-2 levels. The histopathological evaluation of the rats' knee joints showed that rats treated with CLC patches showed similar near-normal structures, regular smooth cartilage surfaces, and dense bone joint lumens when compared to those of the control group. These results show that the CLC preparation had good protective and therapeutic effects on bone tissue damage caused by MIA-OA (Table 4).

**TABLE 4:** The effect of alkaloid active ingredients combined with carriers on OA cartilage repair.

Research (author & year)	BDEs	Carrier	Gene expression		Cartilage repair effects		Other evaluations	Research conclusion
			Inhibition	Promote	Imaging evaluations	Tissue sections		
Chen et al. (2012)	BRU	MH	IL-1β, TNF-α		The severity of OA was milder in the BMH group than in the saline group *via* gross pathological observation	The number of fibroblasts was significantly decreased and the integrity of synovium was improved in the BS and BMH groups than in the saline group	X-ray (release profiles of BRU), SEM, FX imaging	BRU-loaded MH can inhibit the expression of TNF- α and IL-1β to protect OA joint
Chen (2015)	SIN	CM/GelMA	IL-1β, ADAMTS5	Col.II, ACAN, LC3	No mention	The combination of CM-SIN and GelMA hydrogel retarded the progression of surgically induced OA, while each of these components alone also had a mild beneficial effect according to the OARSI score	SEM, RT-PCR	SIN combined with scaffolds can improve the progression of surgically induced OA by promoting autophagy
Mohamed et al. (2020)	CLC	MSN	IL-1β, NO, COX-2		No mention	the rats group treated with formula 2 showed nearly normal architecture like normal control with a regular smooth surface	TEM, FTIR, FE-SEM	COL-MSN/hydrogel patch is an effective, safe, and convenient treatment for OA
Chen et al. (2018)	BBR	HA/SA	ADAMTS5, MMPs	Col.II, ACAN, BV/TV, Wnt/β-catenin, LC3	The defect surface became smooth in the group of scaffold + BER, by general observation, and the results of the micro-CT scans demonstrated that much more calcified tissue was produced at 4 and 8 weeks post-surgery in the defect regions implanted with the scaffold in combination with BER than in those implanted with the scaffold without BER	The OA mice treated with BER showed better cartilage surfaces with cracks and a markedly lower OARSI score compared to that of the untreated OA mice		BER combined with HA/SA can activate Wnt signaling pathway, repair subchondral bone, and promote autophagy to protect the cartilage
Zhou et al. (2015a)	BBR	CNs	Bax, caspase-3	Bcl-2	The cartilage damage in the OA + BBR–loaded CNs group was significantly reversed by general observation	Mankin scores revealed that BBR-loaded CNs treatment antagonized a stronger effect on the amelioration of cartilage damage		BBR-loaded CNs further showed anti-apoptosis activity in the treatment of OA
Zhou et al. (2017)	BBR	CMs	ADAMTS5, MMPs, caspase-3		No mention	No mention		BBR-CMs showed enhanced anti-apoptotic and chondroprotective effects on the treatment of OA

SEM, scanning electron microscopy; LC3, 1A/1B-light chain 3; RT-PCR, real-time polymerase chain reaction; TEM, transmission electron microscopy; BMSCs, bone marrow–derived MSCs; MMx, medial menisci resection; MH, chitosan–glycerol–borax microsphere/thermally responsive hydrogel; CM/GelMA, chitosan microspheres and photo-cross-linked gelatin methacryloyl hydrogel; MSN, mesoporous silica nanoparticles/hydrogel; HA/SA, hyaluronate and SA scaffold; CNs, chitosan nanoparticles; CMs, chitosan microspheres; BS, brucine solution; BER, berberine.

## 5 Others

### 5.1 Ginger Extract

Ginger is the rhizome of *Zingiber officinale* Roscoe, and its extract can inhibit the mitochondrial pathway of cell apoptosis (Hosseinzadeh et al., 2017). When inflammation causes apoptosis of articular chondrocytes in OA, the value of apoptosis mitochondrial pathway marker Bax/Bcl-2 increases (Lee et al., 2004). Numerous studies have shown that the treatment of OA with ginger extract (GINE) is effective and safe (Bliddal et al., 2000; Leach and Kumar, 2008; Bartels et al., 2015; Rondanelli et al., 2020). Experimental studies found that 0.01, 0.5, 1, 5, 10, 25, 50, and 100 μg/ml of GINE was nontoxic to C28I2 chondrocytes, and 5 and 25 μg/ml of GINE can reduce the ROS production, upregulate the expression of antioxidant enzymes GPx-1, GPx-3, GPx-4, and SOD1, reduce the value of Bax/Bcl-2, inhibit the activation of caspase-3, inhibit the apoptosis mitochondrial pathway signal stimulated by IL-1β, and reduce apoptosis of human C28I2 chondrocytes (Hosseinzadeh et al., 2017; Li et al., 2020). However, the oral administration of GINE can cause gastrointestinal reactions, poor absorption, rapid metabolism, and the elimination of the active compound, and GINE itself has low bioavailability. Therefore, several studies have suggested that its transdermal administration is safer than oral (Amorndoljai et al., 2015).

There is only one clinical study that used GINE combined with a cartilage tissue engineering carrier. Amorndoljai et al. (2015) prepared a topical liniment by combining GINE and nanostructured lipid carrier (NLC). About 60 KOA patients (aged 50–75 years) were included afterward and applied this liniment three times per day. After 12 weeks of treatment, the GINE nanoparticles significantly improved the patients' overall assessment, knee pain, symptoms, daily activity, physical activity, and quality of life, and their Knee Injury and Osteoarthritis Outcome Score (KOOS), Index of Severity for Osteoarthritis (ISOA), and Patient's Global Assessment (PGA) results were statistically significant (*p* < 0.05). There were no safety problems or adverse events.

### 5.2 Cordycepin

Cordycepin (3′-deoxyadenosine) (COR) is a nucleoside analog isolated from *Cordyceps militaris* (L.) Fr., which occupies a notable place in traditional medicine. In recent years, many studies have verified that COR has anticancer, antiangiogenesis, antiaging, and other pharmacological effects (Khan and Tania, 2020). It has also been found that COR can induce cell apoptosis and autophagy. Moreover, a recent study has shown that COR significantly inhibited the production of PGE_2_ and NO and decreased the production of MMP13, IL-6, iNOS, and COX-2 in OA chondrocytes induced by IL-1β (Hu et al., 2014). These clues suggest that COR inhibits TGF-β activity, induces autophagy, and prevents cartilage degradation to protect the chondrocytes (Ying et al., 2014; Ashraf et al., 2019; Tao et al., 2020). It is suggested that COR may be a potential candidate drug for the prevention of OA. However, few studies have evaluated its efficacy in the treatment of OA (Xia et al., 2017).

There is only one animal study that used COR combined with a cartilage tissue engineering carrier. Xia et al. (2017) investigated the synergistic effects of hydrogel and COR on the promotion of chondrocyte autophagy, both *in vivo* and *in vitro*. All of the CMs-encapsulated COR (CM-COR) was released in phosphate-buffered saline within 72 h. COR decreased the mRNA expression of MMP13 and ADAMTS5 and increased the mRNA expression of COL2A1 and ACAN. In addition, COR significantly increased the expression of LC3-positive chondrocytes in cartilage tissue, suggesting that COR can activate autophagy to prevent IL-1β–induced cartilage degradation. After receiving CM-COR + hydrogel, cartilage degeneration decreased, the OARSI score reached 3.4 ± 0.3, and the progression of OA was delayed to the greatest extent.

### 5.3 Tetramethylpyrazine/Ligustrazine

Tetramethylpyrazine (TMP), also called ligustrazine (LIG), is a botanical extract component separated from *Conioselinum anthriscoides* “Chuanxiong,” which has strong anti-inflammatory (Wei et al., 2016; Chen et al., 2017) and cartilage protection properties (Ju et al., 2010). In recent years, TMP has proven to be an effective candidate for the treatment of OA (Zhang et al., 2018) as it can reduce IL-1β–induced GAG degradation, MMP3 mRNA expression, and IL-1–induced cartilage and chondrocyte degeneration, thereby improving chondrocyte activity and inhibiting their apoptosis. ROS and the apoptotic mitochondrial pathways influence articular cartilage degeneration in OA (Kim and Blanco, 2007; Musumeci et al., 2015). Furthermore, TMP scavenges cytotoxic ROS to maintain the mitochondrial membrane potential and downregulate caspase-3 activity (Ju et al., 2010). However, TMP is quickly erased from the articular cavity after IA injection, so multiple injections are required to maintain the curative effect, which may lead to the inflammation and infection of local tissues. Reducing the number of injections is the key to increasing patient compliance (Zhang et al., 2018).

There are three animal studies that used TMP/LIG combined with cartilage tissue engineering carriers. Zhang et al. (2018) evaluated the curative effects of TMP microspheres on joint swelling and histological analysis in a papain-induced OA rat model. The drug loading of freeze-dried microspheres (81.36 ± 1.15%) was greater than that of vacuum-dried microspheres (8.22 ± 0.19%). The freeze-dried microspheres had a particle size of about 10 µm and excellent sustained release properties, effectively able to extend the release time of the drug in the articular cavity to 30 days (4.7 times more than the TMP solution). TMP microspheres were also superior to the TMP solution in improving the range of motion and swelling of OA joints and promoting the repair of cartilage defects. TMP microspheres showed powerful advantages in reducing drug dose, limiting injection times, and improving efficacy. Two other similar studies showed that a combination of nanoparticles and LIG could improve bioavailability and targeting, and effectively reduce synovial MMP levels and NF-κB expression upstream of the NF-κB signaling pathway, thereby alleviating KOA and reducing adverse reactions (Ji et al., 2021). The combination of TMP and nanoparticles can lengthen the retention in the articular cavity, enhance the concentration of TMP, and improve the anti-inflammatory effects while avoiding systemic toxicity (Li et al., 2021).

### 5.4 Andrographolide

Andrographolide (AG) is a major plant extract of *Andrographis paniculata* (Burm. f.) Nees, which is known for its antioxidant and anti-inflammatory effects (Burgos et al., 2020; Kulsirirat et al., 2021a). A double-blind, randomized, placebo-controlled study also found AG (300 and 600 mg/day) to be effective and safe in relieving pain in patients with mild to moderate KOA (Hancke et al., 2019). The pharmacological mechanism may be as follows: AG suppresses synovial inflammation *via* the regulation of TNF-α receptor 2 (TNF-R2) trafficking (Wang et al., 2021b); activates the Keap1-Nrf2-ARE pathway, and increases cell proliferation inhibited by H_2_O_2_ and antioxidant enzyme activity to alleviate the oxidative stress damage of chondrocytes (Li et al., 2018); specifically, AG can promote the osteogenesis and chondrogenesis of human suprapatellar fat pad–derived MSCs (Kulsirirat et al., 2021b), and it inhibits the activation of NF-κB and MMP13 expression stimulated by IL-1β in the chondrocytes (Ding et al., 2013; Chen et al., 2020b). However, the low water solubility and high lipid solubility of AG reduce its bioavailability and thus limit its oral absorption (He et al., 2021). Therefore, a continuous delivery system is needed to maximize the pharmacological benefits of AG.

There are two animal studies that used AG combined with a cartilage tissue engineering carrier. He et al. (2021) used an OA rat model to verify the anti-inflammatory and cartilage protection ability of AG combined with mesoporous silica nanoparticles (AG-MSNs). The results showed drug encapsulation efficiency of 43.39 ± 0.33% and drug loading capacity of 22.38 ± 0.71%, while at pH = 5.6, AG could be released continuously for 48 h with an accumulated release rate of about 80%. AG-MSNs prevented the IL-1β–mediated upregulation of MMP3 and MMP13 (64.66 and 67.33%). AG and AG-MSNs showed remarkable anti-inflammatory properties, decreasing the damage to chondrocytes stimulated by IL-1β. It is noteworthy that pH-responsive polyacrylic acid (PAA)–loaded Ag-MSNs was superior to AG-MSNs in the OARSI score, cartilage protection effects, and anti-inflammatory effects. In addition, Kulsirirat et al. (2021a) showed that combined use of a gelatin-based hydrogel with AG-NPs significantly extended the duration for more than 1 month. Unfortunately, only the *in vivo* sustained release time of the drug delivery system was reported, and no analysis of cartilage defect repair was performed.

### 5.5 Celastrol

Celastrol (CSL) is a pentacyclic triterpenoid extracted from *Tripterygium wilfordii* Hook. f., which has been widely used to treat RA (Song et al., 2019), systemic lupus erythematosus (Xinqiang et al., 2020), and cancer (Wang et al., 2007). Previous studies have shown that CSL can block IL-1β and TNF secretion in OA animals and eliminate the infiltration and proliferation of immune cells, thereby preventing cartilage and bone defects (Liu et al., 2020b). In different types of cells, CSL can inhibit IKK complex phosphorylation IκBα, one of the key steps of NF-κB activation (Jin et al., 2020a). The NF-κB signaling pathway is a typical signaling pathway involved in the development of OA pathobiology. The NF-κB transcription factor can induce cartilage degradation and promote the secretion of a variety of degradation enzymes such as MMPs and ADAMTS, which play a key role in the degradation of ECM structural proteins (Feng et al., 2020). However, the clinical application of CSL is limited due to its systemic toxicity and poor bioavailability caused by its low water solubility (13.25 ± 0.83 μg/ml at 37°C) (Wang et al., 2007). There is only one animal study that used CSL combined with a cartilage tissue engineering carrier. Jin et al. (2020a) verified an anti-inflammatory therapy against KOA through the IA injection of hollow mesoporous silica NPs loaded with CSL. the NPs largely improved the dissolution rate of CSL to 73.99% (from 12.96%). When pH = 6, the release of CSL was triggered, exerting an anti-inflammatory effect, cartilage protective effect through the prevention of COL2 degradation, and downregulation of MMP3 and MMP13 expression.

### 5.6 11-Keto-β-Boswellic Acid

Numerous studies on the pharmacological properties of *Boswellia serrata* Roxb. gum-resin extract 11-keto-β-boswellic acid (KBA) have shown its therapeutic effects on inflammatory diseases such as OA, RA, inflammatory bowel disease, and cancer (Bairwa and Jachak, 2016; Efferth and Oesch, 2020). KBA downregulates pro-inflammatory cytokines such as TNF-α, IL-1, IL-2, IL-4, IL-6, and IFN-γ and increases the phagocytosis of macrophages. The inhibitory effect of KBA on 5-lipoxygenase could lead to a decrease in leukotriene production. Various chronic inflammatory diseases are associated with increased leukotriene activity (Ammon, 2010; Efferth and Oesch, 2020). However, the pharmacological activity of KBA is limited by its low oral bioavailability, low water solubility and high Phase I metabolism (Bairwa and Jachak, 2016). There is only one animal study that used KBA combined with a cartilage tissue engineering carrier. Bairwa and Jachak (2016) developed an oral preparation of KBA nanoparticles (KBA-NPs) to improve its bioavailability and anti-inflammatory activity. The particle size was 152.6 nm, the polydispersity index was 0.194, the encapsulation rate was 79.7%, and the cumulative release of KBA was 61.5% at 72 h. Compared with KBA suspension (34.9%), the inhibition rate of KBA-NPs on rat foot swelling at 5 h was 60.8%, and the bioavailability and anti-inflammatory activity of KBA-NPs were increased by seven times and 1.7 times, respectively. The increased anti-inflammatory activity of KBA-NPs may be due to its increased bioavailability. However, the study did not report the effects of the administration system on cartilage defect repair (Table 5).

**TABLE 5:** The effect of other effective components of traditional Chinese medicine combined with carriers on OA cartilage repair.

Research (author & year)	BDEs	Carrier	Gene expression		Cartilage repair effects		Research conclusion
			Inhibition	Promote	Imaging evaluations	Tissue sections	
Amorndoljai et al. (2015)	GINE	NLC			No mention	No mention	GINE nanoparticles alleviated joint pain, improved symptoms of KOA
He et al. (2021)	AG	MSNs-PAA	IL-1β, MMPs	Col.II, GAG, ACAN	AG@MSNs-PAA displayed minimal changes in cartilage compared to the other three ACLT groups by general observation	Matrix vertical fissures, thinner cartilage as well as minor surface destabilization were observed	AG@MSNs-PAA can effectively inhibit the development of OA
Kulsirirat et al. (2021a)	AG	PLGA-NPs			No mention	No mention	AG-NPs-PLGA can prolong the duration to improve the therapeutic efficacy
Xia et al. (2017)	COR	CM-HAMA	IL-1β, MMPs, ADAMTS5	Col.II, LC3, ACAN	No mention	In comparison with IL1-β–treated cartilage, cartilage that was simultaneously treated with IL1-β and COR exhibited more Safranin-O–positive proteoglycan	COR improves cartilage matrix degradation by inducing autophagy
Zhang (2017)	TMP	PLGA-Ms			No mention	The cartilage damage was improved in the treatment group compared to the untreated OA model; the cartilage layer recovered integrity and chondrocytes arranged in normal	IA injection of TMP microspheres can effectively relieve inflammatory symptoms
Jin et al. (2020a)	CSL	MSNs-Cs	IL-1β, IL-6, TNF-α, MMPs, NF-κB		A profoundly reduced knee swelling and improvement in synovial inflammation and cartilage integrity were demonstrated in the CSL@HMSNs-CS group by MRI	A dramatic improvement in pathological changes, such as smooth cartilage surface, undulating tide line, and cartilage thickness was observed in the CSL@HMSNs-CS group	HMSNs-Cs can improve the solubility and bioavailability of CSL
Bairwa and Jachak, 2016	KBA	PLAG-NPs			No mention	No mention	The bioavailability and anti-inflammatory activity of KBA in KBA-NPs were increased

AG@MSNs-PAA, andrographolide-loaded mesoporous silica nanoparticles with pH-responsive PAA; PLGA, poly(lactic-co-glycolic acid); PLGA-NPs, PLGA nanoparticle–gelatin hydrogel; CM, chitosan microspheres; HAMA, HA methacrylate; PLGA-Ms, PLGA microspheres; MSNs-Cs, mesoporous silica nanoparticles-chitosan; PLAG-NPs, PLAG–polyvinyl alcohol nanoparticles; HMSNS-Cs, Hollow mesoporous silica nanoparticles capped with chitosan; CSL@HMSNs-CS, celastrol loaded HMSNS-Cs.

## 6 A Summary of the Application of Botanical Drug Extracts and Carriers in the Repair of Cartilage Defects

### 6.1 Preparation of Carriers

Scaffolding is of great significance in cartilage tissue engineering. It is worth noting that electrostatic spinning technology and 3D printing technology are the hot spots in cartilage tissue engineering at present.

Ming et al. (2018) prepared PLA/gelatin 3D nanofiber sponge loaded with resveratrol (RES) using the electrostatic spinning technology reported by Chen (Chen et al., 2016c; Chen et al., 2016d). First, 0.1 g of PLA and 0.75 g of gelatin were mixed in 9 ml of 1,1,1,3,3,3-hexafluoro-2-propanol with a mass ratio of 1:5 of gelatin to PLA and stirred at room temperature for 5 h to prepare the PLA/gelatin solution. RES was then added, and the mixture was dissolved in 0.1 ml of ethanol and stirred until uniform. The mixed solution was then loaded into a stainless-steel needle at a flow rate of 3 ml/h at an applied voltage of 15 kV. The distance between the needle and the collector was 10 cm. All the collected PLA/gelatin nanofiber membranes were placed in a vacuum overnight to remove the residual solvents. The nanofiber membranes prepared as above were cut into small pieces (0.5 × 0.5 cm), then 1 g of the membranes was weighed and added to 100 ml of tert-Butanol and dispersed by a homogenizer at a speed of 10,000 r/min for 20 min. The dispersed nanofibers were then poured into a 24-well cell culture plate and freeze-dried for 24 h, and the freeze-dried 3D nanofiber sponge was heated in air at 190°C for 2 h for cross-linking in order to obtain the final scaffold.

Kann et al. (2016) dissolved 2 g of SA and 6 g of gelatin (SA/gel, 1:3 ratio) in 50 ml of ddH_2_O to preprocess the colloid solution. The 3D printer parameters were set as follows: printing pressure of 0.20–0.40 MPa, line spacing of 0.3–1.2 mm, printing diameter of 210–510 μm, printing speed of 4–16 mm/s, and printing temperature of 200°C, kept constant throughout the printing process. The printed SA/gel composite scaffold was promptly soaked in 5% CaCl_2_ for 30 min for rapid solidification, then soaked in 2% glutaraldehyde for 24 h for cross-linking. Residual glutaraldehyde was removed *via* l-glutamate treatment, and the scaffold was freeze-dried. In addition, Zhu et al. (Zhu et al., 2020) first prepared acellular cartilage polyethylene diacrylate (PEGDA)/ECM hydrogel using the optical cross-linking method, then prepared PEGDA/ECM 3D ink *via* dynamic projection stereophotography. Moreover, many preparation methods for drug-loading material scaffolds have been widely used in cartilage tissue engineering, such as hydrogel prepared by chemical or photo cross-linking (Goldring and Goldring, 2016) (Rai et al., 2017), aerogel prepared *via* the sol–gel method (Xia et al., 2014), membrane hydroliposomes (Wang et al., 2013) and NPs prepared by ion cross-linking (Miller et al., 2016).

### 6.2 Combination of Botanical Drug Extracts and Carriers

Through an analysis of the relevant literature, the combination of extracts and carriers is classified into two categories according to the different periods of ingredient addition: reactive combination and cell culture.

I. Reactive combination: This is the method chosen for many studies (Charlier et al., 2019; Jin et al., 2020a; Oliveira Silva et al., 2020). The combination is carried out through a series of chemical reactions between the BDEs and the carrier material preparation. For example, Sheu et al. (Goldring and Goldring, 2016) dissolved RES and HMDI in a dry tetrahydrofuran solution, slowly heated it to 50°C for 50 h, then gradually added H_2_O to the reaction mixture (over 30 min) to generate RES-HMDI-NH_2_. Oxi-hyaluronan (OXI-HA) solution was then mixed with the RES-HMDI-NH_2_ solution to form an Oxi-HA-RES solution. Finally, the oxidized HA/RES hydrogel was prepared. II. Cell culture: Considering the cytotoxicity of some ingredients, this method is more suitable for confirming safe and nontoxic BDEs, so there are fewer studies. Kann et al. (2016) incubated cells in a normal medium for 24 h. After the cells had adhered normally, they took the medium out and replaced it with a medium containing ICA. After incubation for 1, 3, 5, and 7 days, they observed the cell proliferation. Finally, cells cultured with the extracts were planted on the carrier.

A total of 31 reports were included in this review, among which 12 were not studied in animal models, 17 were tested by IA injection, one was tested by intraperitoneal injection, and the remaining one was a clinical trial. In the selection of carriers, nanoparticles, microspheres, hydrogels, and 3D printed carriers are chosen. It is noteworthy that due to the pathological relationship between low pH and OA, acid-sensitive hydrogels prepared from mesoporous silica are superior in drug release, anti-inflammatory, and cartilage defect repair properties. The combination of cartilage tissue engineering materials improves the bioavailability of BDEs, prolongs the time of drug action, and alleviates adverse reactions.

We can find that almost all BDEs that can be used to treat OA have anti-inflammatory and antioxidant effects. Among them, CUR, BBR, and HON can not only protect cartilage but also promote the formation of subchondral bone, which is more suitable for the treatment of patients with subchondral bone damage in the advanced stages of OA. In addition, for BRU and CLC, we need to develop more appropriate administration systems to reduce their toxicity. Aside from anti-inflammatory and cartilage protective effects, Hes, SIN, and COR can also induce autophagy *in vivo* to mitigate the degradation of the cartilage matrix. At present, RES and CUR are the most studied BDEs, but they are still in the animal experiment stage. Further studies are required to confirm our hypotheses on how to further improve efficacy and whether they are clinically feasible (Table 6).

**TABLE 6:** A summary of key advancements in the field.

Category	Key advancements	References
Herbal monomer	CUR and RES are the most used herbal monomers in cartilage repair, and the optimal concentration of RES is 114.281 mol/L	Ming et al. (2018)
	Coencapsulated RES and CUR in lipomolecular nanocapsules, improved the photostability of RES and the *in vitro* antioxidant activity of both polyphenols	Coradini et al. (2015), Kann et al. (2016)
	CUR, BBR, and HON can not only protect cartilage but also promote the formation of subchondral bone	Chen et al. (2012), Yeh et al. (2015), Zhu et al. (2020)
	Both BRU and CLC have severe biotoxicity, pay attention to test its safety	Chen et al. (2012), Mohamed et al. (2020)
	Hes, SIN, and COR can induce autophagy *in vivo* to improve the degradation of the cartilage matrix	Chen (2015), Xia et al. (2017), Ouyang et al. (2019)
Carriers	GD2(CO3)3@PDA—a gadolinium (III) (Gd3+) containing MRI contrast agent coated with PDA, a good drug carrier for the targeted delivery of OA drugs to lesion sites	Ouyang et al. (2019)
	Prepared a high porosity 3D scaffold based on cellular responsive polymer ink SA and gelatin (SA-gel, 1:3)	Kankala et al. (2018)
	The use of PLA can enhance the bioabsorbability and drug carrying capacity of nanoparticles	Kamel et al. (2019)
	Microfluidic drug screening device can effectively screen the drug concentration required for cell culture	Ming et al. (2018)
	The thermosensitive composite hydrogel prepared with chitosan as temperature-sensitive material can change the distribution of drugs in the joint cavity, increase the concentration of drugs in the joint cavity, and delay the retention of drugs in the target region	Chen et al. (2012)
	PAE can be used to achieve controlled drug release at low PH, and the positively charged PAE can interact with the GAG in the cartilage by electrostatic interaction to achieve targeted drug delivery	Kang (2019)
	AG@MSNs-PAA nanoplatform formed by modified MSNs and pH-responsive PAA is favorable for sustained release in the OA environment	Jin (2020), He et al. (2021)

PDA, polydopamine; AG@MSNs-PAA, andrographolide-loaded mesoporous silica nanoparticles with pH-responsive PAA.

## 7 Conclusion

This article reviews the role of BDEs combined with biomaterial carriers in the treatment of OA and the repair of cartilage degeneration in recent 10 years. Many studies have shown that BDEs play an anti-inflammatory, anti-oxidation, and cartilage protection role by inhibiting the expression of pro-inflammatory factors (IL-1β, IL-6, and TNF-α) and matrix degrading enzymes (MMP1, MMP3, MMP13, and COX-2), and removing oxidative stress substances such as NO, ROS, and H_2_O_2_. They can also promote the repair of cartilage degeneration and delay the progress of OA by regulating chondrocyte autophagy. Some BDEs (e.g. RES, CUR, ICA, and Hes) can also promote the production of COL2 in the cartilage ECM, thus promoting the production of hyaline cartilage (instead of fibrocartilage) at the defect site, which is very beneficial to the recovery of normal joint function. In addition, a few BDEs, such as CUR, HON, and BBR, play an osteogenic role through OPG/RANKL and Wnt/β-catenin signaling pathways and promote the repair of subchondral bone defects, so they are more suitable for the injury of subchondral bone involved in late OA. At present, the clinical drugs used for OA treatment are mostly limited to symptomatic treatment to relieve joint pain and improve joint function. Comparatively, BDEs can not only improve the joint function and eliminate clinical symptoms but also promote the repair of damaged cartilage in multiple ways and targets, and delay the progress of OA.

Since the progression of OA is a long-term, chronic process, the patients need long-term administration. However, oral administration has poor bioavailability and long-term oral administration can cause some serious adverse reactions. On the contrary, local IA injection or drug carrier implantation greatly improves drug utilization and reduces systemic response. Compared with local injection of pure BDEs, the sustained-release effect of the biomaterial carrier can enhance the retention time of the drug in the joints and enable the drug to function stably for a long time. So how to choose IA injection or drug carrier implantation? The attributes of OA determine a long-term treatment and delaying the progress of OA is the focus of the current clinical treatment. In combination with the included studies, it is more appropriate to select IA injection of drug-carrying biomaterial carriers (hydrogels, nanoparticles, microspheres, etc.) when cartilage damage is not serious in the early and middle stages of OA. Because it is easier to perform than surgical treatment, long-term and repeated injection therapy is possible. For the damage involving subchondral bone in the late stage of OA, the drug carrier implantation is recommended. Compared with the current clinical knee replacement for patients with late OA, drug carrier implantation is theoretically more conducive to the recovery of joint function in patients without worrying about prosthetic wear.

However, long-term repeated injections tend to cause local infection and reduce patient compliance. Therefore, the optimal injection cycle, dose, and times of IA injection need further study and confirmation. It is worth noting that IA injection means that the injection has certain fluidity, so how to improve its biological response, targeting, and mechanical properties is a problem that researchers need to further explore. In addition, because of the irregularity of cartilage damage, the degradability of drug carrier, the biomechanical characteristics of local knee joints, and the different structures of cartilage layers in OA patients, the structural distribution of each layer, the optimal degradation rate, the compatibility and adhesion between the carriers and surrounding tissues need further research during stent fabrication.

It is found in the included studies that almost all researchers chose chondrocytes rather than MSCs in the selection of seed cells. However, many studies have confirmed that MSCs as seed cells are more suitable for cartilage repair. Then, whether BDEs can promote or induce the differentiation of MSCs into chondrocytes and the related mechanism of action are also questions worthy of consideration. In addition, because the mechanism of action of most BDEs (some having certain toxicity) has not been fully understood yet, the rejection reaction of the body, as well as the changes in the biological environment, chemical stimulation, and microenvironment in the damaged area, researchers need to further study and confirm the selection and manufacture of materials; the selection, dose, concentration, etc. of BDEs. At present, all *in vivo* animal studies have confirmed the effectiveness and safety of BDEs combined with biomaterial carriers, but there is no related clinical experiment to further verify their effectiveness and safety. Therefore, more relevant studies are needed to address the current problems.
